# Evaluation of the double-zone hemolysis (DZH) test for the detection of livestock-associated methicillin-resistant *Staphylococcus aureus*

**DOI:** 10.1128/spectrum.01102-24

**Published:** 2024-12-10

**Authors:** Javier Latorre-Fernández, Carmen Aspiroz, Idris Nasir Abdullahi, Allelen Campaña-Burguet, Paula Eguizábal, Carmen González-Azcona, Carmen Tenorio, Myriam Zarazaga, Adebayo O. Shittu, Carmen Lozano, Carmen Torres

**Affiliations:** 1 Area of Biochemistry and Molecular Biology, OneHealth-UR Research Group, University of La Rioja, Logroño, Spain; 2 Laboratory of Clinical Microbiology, Hospital Universitario Royo Villanova, Zaragoza, Spain; 3 Department of Medical Laboratory Science, Faculty of Allied Health Sciences, College of Medical Sciences, Ahmadu Bello University, Zaria, Nigeria; 4 Department of Microbiology, Obafemi Awolowo University, Ile-Ife, Nigeria; 5 Institute of Medical Microbiology, University Hospital Münster, Münster, Germany; Icahn School of Medicine at Mount Sinai, New York, New York, USA

**Keywords:** hemolysis, *Staphylococcus aureus*, CC398, LA-MRSA

## Abstract

Livestock-associated methicillin-resistant *Staphylococcus aureus* (LA-MRSA), such as clonal-complex (CC)398, are of clinical relevance due to their multi-drug resistance profiles, adding to the overall burden of MRSA in humans. The objective was to evaluate the double-zone hemolysis (DZH) test as a simple and reliable method for detecting LA-MRSA in the clinical microbiology laboratory. *S. aureus* isolates assigned to CC398 (*n* = 183; 152 MRSA/31 methicillin-susceptible *S. aureus* [MSSA]), CC1 (*n* = 44; MRSA), and other CCs (*n* = 144; 94 MRSA/50 MSSA) were investigated. These isolates were screened for DZH on sheep blood agar plates after incubation at 37°C for 24 h. Identification of the *scn* (human adaptation marker) and *hlb* genes (encoding hemolysin, intact or truncated) was performed by PCR. The positive and negative predictive values (PPV and NPV), sensitivity (SS), and specificity (SP) of the DZH test were determined. The DZH-positive phenotype was observed in 94.7%, 25%, and 6.4% of MRSA-CC398, MRSA-CC1, and MRSA of other lineages, respectively. Moreover, the DZH-positive phenotype was identified in 9.7% of MSSA-CC398 isolates but not in other MSSA lineages. All 164 DZH-positive isolates carried *hlb* intact, and 99.4% was *scn* negative, suggesting an animal origin. Of the 207 DZH-negative isolates, 99.5% was *scn* positive (indicating human adaptation), and 95.2% possessed a truncated *hlb* gene. The PPV/NPV/SS/SP values (in %) of the DZH test were as follows: detection of (i) LA-MRSA-CC398: (87.8/96.1/94.7/90.9); (ii) LA-MRSA-CC398/CC1 *scn* negative: (94.5/100/100/95.8); and (iii) *S. aureus scn* negative: (99.4/99.5/99.4/99.5). The DZH is a reliable strategy to detect and distinguish LA-MRSA in the clinical microbiology laboratory and is recommended as an adjunct diagnostic test.

**IMPORTANCE:**

This study evaluated a simple and reliable phenotypic test that can be very useful in the clinical microbiology laboratory to detect livestock-associated (LA) methicillin-resistant *Staphylococcus aureus* (MRSA) isolates and *S. aureus* of potential animal origin. The proposed double-zone hemolysis test has shown high positive and negative predictive values, sensitivity, and specificity to detect these LA-MRSA clones and *S. aureus* of potential animal origin. Most LA-MRSA clones exhibit resistance to different classes of antibiotics, with unique epidemiological characteristics, and their early detection has public health relevance and patient management.

## INTRODUCTION

*Staphylococcus aureus* is part of the normal microbiota of humans and animals but is also an opportunistic pathogen causing infections of diverse severity (1). *S. aureus* infections are of public health significance, and therapeutic options for methicillin-resistant *S. aureus* (MRSA) are limited as they exhibit different multi-drug resistance profiles (2). *S. aureus* has a broad host range with the capacity for host switching due to the loss or gain of specific virulence factors (3). For instance, the prophage *Sa3*, which carries the immune evasion cluster (IEC) system (which includes the *scn* gene), is mainly found in *S. aureus* lineages of human origin but is absent in isolates of animal origin (4). The IEC system includes genes encoding the staphylococcal complement inhibitor (SCN, *scn*), the chemotaxis inhibitory protein (CHIPS, *chp*), staphylokinase (SAK, *sak*), and some staphylococcal enterotoxin (*sea* and *sep*) genes. Seven IEC types have been identified, and *scn* is included in all IEC types, as it is considered the most important marker of the IEC system (5, 6).

Different MRSA lineages associated with animals have been described, including clonal complex (CC)398, which is prevalent in livestock globally (7, 8). The livestock-associated (LA)-MRSA-CC398 clone was first reported in 2005 in pigs and pig farmers (9). Since then, MRSA-CC398 has been increasingly described in the pig environment and in colonized or infected individuals in contact with pigs (7, 10–12). Also, a high prevalence of MRSA-CC398 in clinical settings is associated with high pig farming density in the surrounding areas where hospitals are located (13, 14). MRSA-CC398 isolates exhibit multi-resistance to many antimicrobial agents, including tetracycline (15, 16). Two clades of *S. aureus* CC398 (human- and animal-adapted) have been described (17) and differentiated by the identification of specific SNPs (18). Strains belonging to the animal-adapted clade are generally MRSA, tetracycline resistant (TET^R^), and lack the *scn* gene (integrated into the IEC system). Conversely, the human-adapted CC398 clade is generally methicillin-susceptible *S. aureus* (MSSA), IEC positive, and tetracycline susceptible (TET^S^) (4). In addition to the LA-MRSA-CC398 clone that causes human infections, other LA-MRSA lineages, such as CC1 (*scn*-negative), have been described as causing human infections in hospitals and especially in areas with high pig farming density (19, 20).

Partial, complete, or no hemolysis on blood agar (BA) plates is a useful tool for bacterial identification in the clinical microbiology laboratory. The *hlb* gene that encodes the toxin β-hemolysin (also known as sphingomyelinase) (21–23) is the preferred integration site for the prophage *Sa3* that includes the IEC system (6, 24). A small and clear zone of hemolysis is generally observed when prophage *Sa3* truncates *hlb* (23). However, a double-zone hemolysis (DZH) with an inner zone of β-hemolysis and a large outer zone of incomplete hemolysis on BA plates has been observed in some staphylococcal isolates (25). The DZH is an uncommon phenotype among clinical *S. aureus* isolates from humans (26, 27). However, *S. aureus* isolates from animals often have this typical DZH (23, 26, 28–30). MRSA-CC398 of the animal-adapted clade usually harbors an intact *hlb* (lack the prophage *Sa3* that carries the IEC system), with an increased expression of *hla* and *hlb* genes (encoding alpha- and beta-hemolysins), and a DZH is observed (23). However, human-adapted *S. aureus* isolates frequently carry a truncated *hlb* gene, and the DZH phenotype is not expressed.

Developing simple and rapid diagnostic methods is essential to guide clinical microbiologists in the prompt therapeutic management of patients and epidemiologic surveillance. In a previous study, the TET^R^ phenotype was a good marker for LA-MRSA detection (13). The present study aims to advance this observation by evaluating the DZH to discriminate between animal- and human-adapted MRSA lineages.

## MATERIALS AND METHODS

### *S. aureus* isolates included in the study

This study comprised 371 *S. aureus* isolates (290 MRSA and 81 MSSA) obtained in previous studies (4, 13, 15, 19, 31–37) of the OneHealth-UR Research Group of the University of La Rioja and re-confirmed as *S. aureus* by matrix-assisted laser desorption/ionization time-of-flight mass spectrometry (MALDI-TOF-MS) (Bruker Daltonik, Bremen, Germany). The clonal lineages based on multi-locus sequence typing (MLST), the staphylococcal protein A gene (*spa*) types, the detection of the IEC gene cluster, and the antimicrobial resistance phenotypes of most of these isolates were determined in previous studies (4, 13, 15, 19, 31–37). The origins of the isolates are as follows: (i) human origin: *n* = 359, including MRSA-CC398 (*n* = 151), MRSA-CC1 (*n* = 44), MSSA-CC398 (*n* = 20), and MSSA and MRSA isolates of other genetic lineages (*n* = 144); (ii) animal origin: *n* = 9, all MSSA-CC398; and (iii) food origin: *n* = 3, one MRSA-CC398 and two MSSA-CC398.

### DNA extraction

The isolates, stored in skimmed milk at −80°C, were streaked on BHI agar and incubated at 37°C for 18–24 h. For the DNA extraction, one colony was suspended in 45 µL of sterile MiliQ water and 5 µL of lysostaphin (1 mg/mL) (Sigma). Then, it was vortexed and incubated at 37°C for 10 min. A 45-µL volume of sterile MiliQ water, 150 µL of Tris-HCl (0.1 M, pH 8), and 5 µL of proteinase K (2 mg/mL) (Sigma) were added to the reaction mixture, vortexed, and incubated at 60°C for 10 min. Finally, it was boiled (100°C) for 5 min and centrifuged at 12,000 rpm for 3 min. The DNA contained in the supernatant was stored at −20°C.

### Characterization of the isolates

The characteristics of the isolates, i.e., *spa*-type, CC, presence of *scn* and phenotypic detection of TET^R^, and *mecA* gene detection in the collection, were determined in some of the isolates as previously reported (6, 16) (this information was already known for most of the isolates from previous studies). Furthermore, all the isolates were screened for *hlb*, as previously described (38). The amplification of a 309-bp fragment of *hlb* (that includes the point of integration of the prophage *Sa3* that carries the IEC system) provided evidence for an intact gene. To ascertain if the *hlb* gene was intact (without the insertion of the prophage *Sa3*) or truncated (with the insertion of the prophage *Sa3*)*,* PCR was also performed to amplify different sections of the *hlb* gene, as previously reported (21).

### Study of DZH

The DZH phenotype was investigated among the *S. aureus* isolates. Briefly, one colony of each isolate was seeded in BA plates containing 5% of sheep blood (bioMérieux), incubated at 37°C for 24 h, and observed for hemolysis. The DZH hemolysis was defined as an inner zone of β-hemolysis (can be absent) and a large outer zone of incomplete hemolysis on BA plates. The diameter of the DZH was observed with transmitted light and measured with a ruler (in mm). An additional incubation period (at 4°C for 24 h) was conducted as previously described (29), and the diameter of DZH was also measured to compare the results with and without the additional incubation period.

### Correlation of DZH with CC and other phenotypic/genotypic features

The positive predictive value (PPV), negative predictive value (NPV), the sensitivity (SS), and specificity (SP) of the DZH test to detect (i) MRSA-CC398, (ii) LA-MRSA-CC398/CC1-*scn* (−), and (iii) *S. aureus* isolates (*scn* negative) of potential animal origin were calculated as previously reported (Table 1) (39).

**TABLE 1:** . Calculation of DZH method characteristics

Test	Target of the DZH test	
	Positive	Negative
DZH+	True positive A	False positive B
DZH−	False negative C	True negative D

The calculation of the predictive value, sensitivity, and specificity of the studied groups (LA-MRSA-CC398, LA-MRSA-CC398/CC1 *scn* [−], and *S. aureu*s *scn* [−]) was performed, using the following equations:


Sensitivity:A/(A+C);Specificity:D/(B+D)

PPV:A/(A+B);NPV:D/(C+D)

#### Whole-genome sequencing

Whole-genome sequencing of one isolate with an atypical DZH phenotype was carried out by Nanopore Oxford Technology (Oxford, the United Kingdom) at the Genomic and Bioinformatic Unit of the Biomedical Research Center of La Rioja (CIBIR, Logroño, Spain). The genomes were analyzed by Geneious Prime 2024.0.7 and compared with the reference strain GD705 obtained from NCBI (GeneBank Accession number: CP019593). The upstream and downstream regions of the IEC genes were illustrated using the EasyFig version 2.2.5 (https://mjsull.github.io/Easyfig/).

### Statistics

Analysis by two-tailed student t-test at 95% CI was performed to determine the mean difference of continuous variables within groups, i.e., the diameter of halo of DZH at two different incubation conditions (37°C for 24 h and 37°C for 24h + 4°C for 24 h) within the individual clonal complex. *P* values < 0.05 were considered statistically significant.

## RESULTS

Table 2 shows the characteristics (CC, methicillin and tetracycline resistance phenotype, and presence or absence of *scn* and *hlb* genes) of the 371 *S*. *aureus* isolates, comprising 290 MRSA and 81 MSSA. Overall, 207 and 164 isolates were *scn* positive and *scn* negative, respectively. In addition, 174 isolates carried an intact *hlb*, and the remaining 197 isolates harbored a truncated *hlb* gene.

**TABLE 2:** Characteristics of the *S. aureus* isolates included in this study^a^

Clonal complex	Number of isolates	Methicillin		Tetracycline		Methicillin^R^ Tetracycline^R^	*scn* gene		*hlb* gene	
		R	S	R	S		Positive	Negative	Intact	Truncated
CC398	183	152	31	156	27	152	37	146	148	35
CC1	44	44	0	44	0	44	33	11	18	26
CC5	41	41	0	10	31	10	38	3	3	38
CC6	1	1	0	0	1	0	1	0	0	1
CC7	1	1	0	1	0	1	1	0	0	1
CC8	21	21	0	9	12	9	17	4	4	17
CC30	2	2	0	2	0	2	2	0	1	1
CC45	10	10	0	10	0	10	10	0	0	10
CC80	2	2	0	2	0	2	2	0	0	2
CC88	1	1	0	1	0	1	1	0	0	1
Others	65	15	50	14	51	14	65	0	0	65
**Total**	**371**	**290**	**81**	**249**	**122**	**245**	**207**	**164**	**174**	**197**

^a^ R, resistant; S, susceptible.

### Interpretation of the DZH phenotypic method

**Fig 1:**
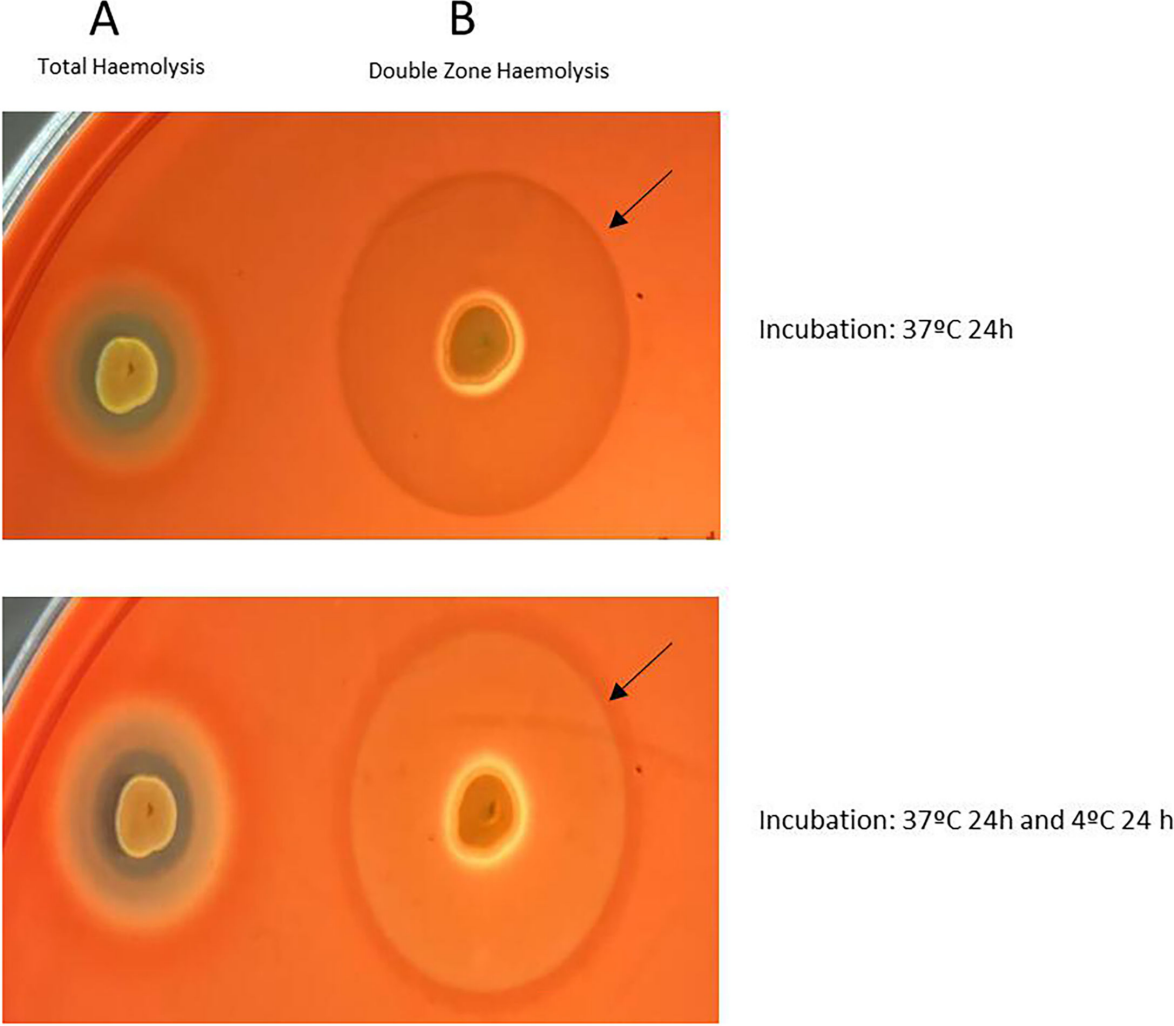
Growth on sheep blood agar of (A) MRSA-CC45 C8969 strain showing beta-hemolysis but not DZH; (**B**) MRSA-CC398 C10412 strain showing DZH. Two incubation conditions were assessed: (i) 37°C for 24 h and (ii) 37°C for 24 h + 4°C for 24 h. The line of partial hemolysis (marked with a black arrow) in DZH-positive strains was more evident after incubation at 4°C. However, the results were clearly visible without additional incubation at 4°C.

The DZH was noted as a small zone of beta-hemolysis (or absent in some cases) but surrounded by a large halo of incomplete hemolysis at 37°C for 24 h (Fig. 1).

The DZH halo (range, media in millimeters) detected among the 164 DZH-positive *S. aureus* isolates under two incubation conditions (i: 37°C for 24 h; ii: 37°C for 24 h + 4°C for 24 h) is shown in Table 3. Only slight differences in the halo diameter were observed between both conditions, with no significant differences. Hence, incubation at 37°C for 24 h is recommended for DZH detection, and the additional 4°C for 24-h incubation is not compulsory. Moreover, it is important to note that the DZH is more visible when the BA plate is under transmitted light.

**TABLE 3:** DZH halo (in millimeters) detected in the collection of 164 *S*. *aureus* isolates analyzed in this study that were positive for this phenotype under two incubation conditions

*S. aureus* lineage	Number DZH-positive isolates	DZH under different incubation conditions			
		37°C for 24 h, mean, SD	37°C for 24 h + 4°C for 24 h, mean, SD	*F* value	*P* value
MRSA-CC398	144	17.4 ± 2.6	18.1 ± 2.5	2.4	0.09
MRSA/MSSA CC398	147	17.6 ± 2.6	18.1 ± 2.6	2.5	0.08
MRSA-CC1	11	16 ± 4.1	16.5 ± 3.6	0.2	0.86
MRSA-CC8	3	19.7 ± 0.6	19.7 ± 0.6	0	1
MRSA-CC5	3	14.3 ± 4.9	14.7 ± 4.6	0.4	0.71

^*a*^ Statistical analysis by one-way Analysis of Variance (ANOVA) within group shows no significant differences.

### Detection of the DZH phenotype

**Fig 2:**
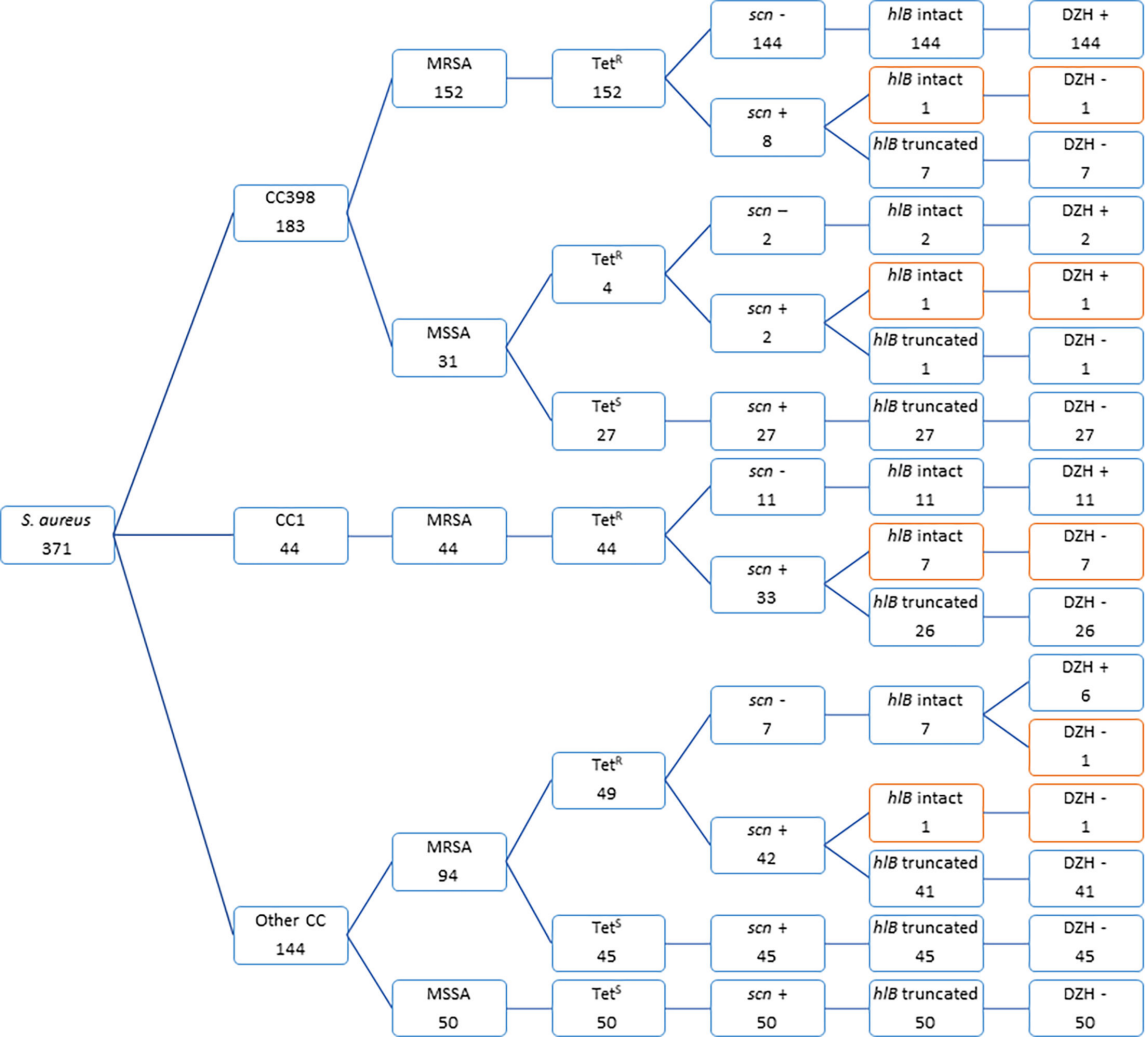
Distribution of the 371 *S*. *aureus* isolates of the different clonal lineages in relation to the MRSA/MSSA phenotype, presence/absence of *scn* gene, type of *hlb* gene (intact or truncated), and results of DZH phenotype. The atypical DZH phenotypes are shaded in red color.

Of the 371 *S*. *aureus* evaluated, the DZH-positive phenotype was observed in 164 isolates comprising MRSA-CC398 (144/152; 94.7%), MRSA-CC1 (11/44; 25%), and MRSA of other lineages (6/94, 6.4%) (Fig. 2). Moreover, the DZH-positive phenotype was identified in isolates assigned with MSSA-CC398 (3/31, 9.7%) but not in 50 MSSA isolates from other lineages. All the 164 DZH-positive isolates were TET^R^ and carried an *hlb*-intact gene, and 99.4% of them (163/164) was *scn* negative. The DZH-positive isolates were largely MRSA-TET^R^ and *scn* negative (161/164, 98.2%), suggesting an animal origin.

Of the 207 DZH-negative isolates, 206 (99.5%) were *scn* positive (suggesting human adaptation) and 197 (95.2%) possessed a truncated *hlb* gene. Moreover, 122 (59%) were TET^S^ (Fig. 2). Also, the DZH-negative phenotype was detected in MRSA-CC398 (8/152, 5.3%), MRSA-CC1 (33/44, 75%), MRSA of other lineages (88/94, 93.6%), and most of the MSSA isolates (78/81, 96.3%).

### Isolates with atypical DZH phenotypes

Eleven isolates displayed an atypical DZH phenotype (Table 4). One DZH-positive and TET^R^ MSSA isolate (C2823) carried an *hlb*-intact gene and was *scn* positive. As the *scn* gene was not inserted in the *hlb* gene, this strain was submitted for whole-genome sequencing. It was observed that the prophage *Sa3* (containing the IEC gene cluster) was inserted outside the *hlb* gene, between the *aes* (encoding an acetyltransferase) and *immA* (encoding a metalloprotease) genes (See Fig. S1). This new IEC insertion site could be due to the modification of the integration site (*attb*) of *hlb*, from “TGTATCCAAACTGG” to “TGTATCCGAATTGG,” leading to the inability of the prophage *Sa3* to insert into the *hlb* gene. Other DZH-negative MRSA isolates (*n* = 10) assigned to CC1, CC8, CC30, and CC398 that showed atypical results were TET^R^, carried an *hlb*-intact gene, and were largely *scn* positive (Table 4 and Fig. 2). Also, the epidemiological data showed that these isolates were of human origin.

**TABLE 4:** Characteristics of the isolates with atypical DZH phenotypes related to the presence/absence of *scn*

DZH	Isolate	MRSA/ MSSA	Tet^R*c*^	*scn^c^*	*hlb* ^*a*^	CC	*spa* type	Origin^*b*^
Positive	C2823	MSSA	+	+	i	CC398	t1197	HCS
Negative	C8924	MRSA	+	−	i	CC8	1148	HCS
Negative	C9135	MRSA	+	+	i	CC398	t1939	HCS
Negative	C8822	MRSA	+	+	i	CC1	t127	HNS
Negative	C8911	MRSA	+	+	i	CC1	t127	HCS
Negative	C8914	MRSA	+	+	i	CC1	t127	HCS
Negative	C8958	MRSA	+	+	i	CC1	t127	HCS
Negative	C9117	MRSA	+	+	i	CC1	t127	HCS
Negative	C9688	MRSA	+	+	i	CC1	t127	HCS
Negative	C8916	MRSA	+	+	i	CC1	t2207	HCS
Negative	C9035	MRSA	+	+	i	CC30	t665	HCS

^*a*^ i, intact.

^*b*^ HCS, human clinical sample ; HNS, human nasal sample.

^*c*^ Symbol "+" means that the strain showed the indicated characteristic and symbol "-" means that it did not show the indicated characteristic.

### Analysis of DZH test to detect animal-associated *S. aureus* lineages

The sensitivity and specificity of the DZH test to detect LA-MRSA-CC398 isolates were very high (>90%) (Table 5). The NPV was >90%, but the PPV was 87.8%. Moreover, the sensitivity, specificity, PPV, and NPV of the DZH test to detect *scn*-negative LA-MRSA (CC1 and CC398) isolates were even higher (>94%). Overall, the DZH phenotype is a strong marker of *scn-*negative isolates and LA-MRSA (CC1 and CC398), while DZH-negative isolates are associated with human-associated lineages.

**TABLE 5:** Positive and negative predictive values, sensitivity, and specificity of the DZH test for detection of livestock *S. aureus* isolates^*a*^

Target of DHZ test	PPV	NPV	Sensitivity	Specificity
LA-MRSA-CC398	87.8%	96.1%	94.7%	90.9%
LA-MRSA-CC398/CC1 *scn* (−)	94.5%	100%	100%	95.8%
*S. aureus* isolates *scn* (−)	99.4%	99.5%	99.4%	99.5%

^*a*^ CC, clonal complex; DZH, double-zone hemolysis; LA-MRSA, livestock-associated methicillin-resistant *S. aureus*; PPV, positive predictive value; NPV, negative predictive value.

## DISCUSSION

MRSA-CC398 is an important lineage in livestock animals, with increasing reports of human infections. Since its discovery, MRSA-CC398 has rapidly emerged as a significant cause of human infections and is mainly associated with livestock exposure. Moreover, MRSA-CC398 exhibits in general wide multi-drug resistance phenotypes that affect diverse families of antibiotics, in addition to beta-lactams (16). The increasing prevalence of this lineage has been observed, particularly in hospitals located in areas with high pig density (13). The prompt identification of MRSA-CC398 and other associated LA-MRSA could assist in taking appropriate epidemiological measures and the clinical management of colonized or infected patients or personnel in areas with high pig farming density.

Most LA-MRSA-CC398 identified in animals or causing human infections lack the *scn* gene (12, 40) and have an intact *hlb* gene and the corresponding DZH phenotype. In this study, the DZH method exhibited high PPV and NPV with high sensitivity and specificity for identifying LA-MRSA-CC398. Furthermore, the DZH test showed even higher values in detecting *scn-*negative LA-MRSA CC398/CC1 isolates. The expression of DHZ has been described in LA-MRSA-CC398 and attributed to the absence of the IEC gene cluster and the overexpression of *hla* and *hlb* (23). Our results support this hypothesis, indicating that this phenotypic method is useful to identify MRSA-CC398 of the animal-adapted clade.

The IEC system (with *scn*) is integrated into the *hlb* gene, which explains our observation that most (206/207, 99.5%) of the *scn*-positive isolates were DZH negative. The impact of prophage *Sa3* carriage on the hemolytic potential of *S. aureus* CC398 has been previously studied, as it mainly affects human-derived erythrocytes (41). However, one *scn*-positive but *hlb*-intact MSSA isolate (C2823) was DZH positive. WGS revealed that the IEC gene cluster was inserted in another region of the genome due to the modification in the integration site, leading to the expression of the DZH phenotype. It has been observed that apart from *hlb*, prophage *Sa3* can integrate into other regions of the genome in LA-MRSA-CC398 due to variations that disrupt the phage attachment site but not the expression of β-hemolysin, as observed in our C2823 strain (21, 42–44). The DZH-positive/*scn*-positive isolate was an MSSA-CC398 nasal isolate obtained from a non-infectious carrier (31). Also, one MRSA-CC8 isolate (C8925) (*scn*-negative, *hlb*-intact) was DZH negative. A study indicated that DZH is not only associated with an intact *hlb* gene but with an overexpression of other hemolysins (23). Moreover, nine MRSA isolates exhibited an atypical phenotype (DZH negative, *scn* positive, and *hlb* intact). These atypical characteristics are candidates for further genomic analyses to understand their mechanisms.

Overall, 360 of 371 isolates (95.2%) confirmed our hypothesis. The detection of DZH on BA plates is a reliable phenotypic marker in detecting animal lineages of *S. aureus*, particularly LA-MRSA-CC398 and MRSA-CC1. We acknowledge the overrepresentation of LA-MRSA-CC398 in this study, and a prospective study is planned to validate the method. Nevertheless, this phenotypic marker could be useful in a clinical microbiology laboratory. An advantage of this method is that the DZH phenotype is easy to perform, and results are available earlier than others, e.g., the detection of tetracycline resistance (15). However, both phenotypic markers are complementary, and a link to the epidemiological information of the environment (e.g., high pig farming density in the area) (13) could improve the precision of this method. Most clinical *S. aureus* isolates from humans with *scn* positive and *hlb* truncated will exhibit a DZH-negative phenotype. The awareness and observation of the DZH phenotype on BA in the clinical microbiology laboratory could elicit a prompt response for effective patient management and epidemiological surveillance, particularly in high pig farming and resource-limited settings.

## Data Availability

The complete sequence of strain S. aureus C2823 was submitted to GenBank accesion number CP171741

## SUPPLEMENTAL MATERIAL

Figure S1 spectrum.01102-24-s0001.tif Supplementary Figure S1. IEC insertion site in: a) C2823 isolate that presented an atypical DZH phenotype; b) CP019593 isolate in which IEC truncates the hlb gene.

ASM does not own the copyrights to Supplemental Material that may be linked to, or accessed through, an article. The authors have granted ASM a non-exclusive, world-wide license to publish the Supplemental Material files. Please contact the corresponding author directly for reuse.
