# Supplementary figures and images for: Evaluation of the double-zone hemolysis (DZH) test for the detection of livestock-associated methicillin-resistant Staphylococcus aureus
Source: Microbiol Spectr. 2024 Dec 10;13(1):e01102-24. doi: 10.1128/spectrum.01102-24 (PMC11705798; doi:10.1128/spectrum.01102-24)

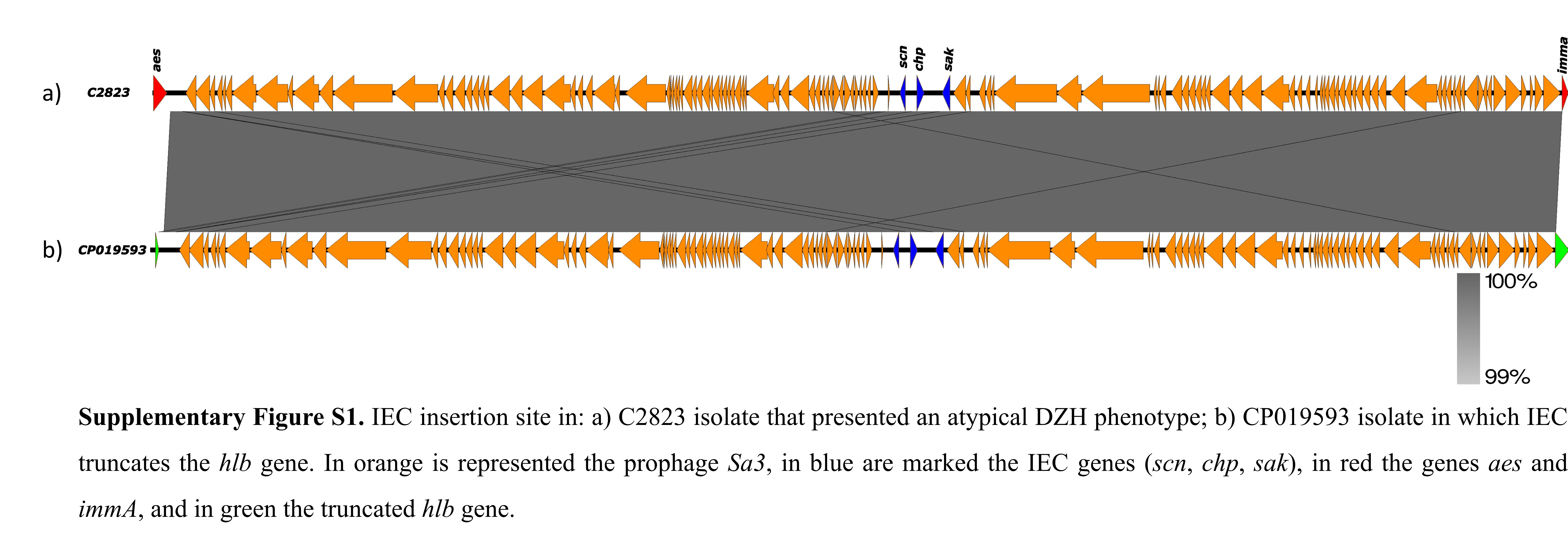

Supplement: Figure S1 — Supplementary Figure S1. IEC insertion site in: a) C2823 isolate that presented an atypical DZH phenotype; b) CP019593 isolate in which IEC truncates the hlb gene. [file spectrum.01102-24-s0001.tif]
